# An Improved In-house MALDI-TOF MS Protocol for Direct Cost-Effective Identification of Pathogens from Blood Cultures

**DOI:** 10.3389/fmicb.2017.01824

**Published:** 2017-09-28

**Authors:** Menglan Zhou, Qiwen Yang, Timothy Kudinha, Liying Sun, Rui Zhang, Chang Liu, Shuying Yu, Meng Xiao, Fanrong Kong, Yupei Zhao, Ying-Chun Xu

**Affiliations:** ^1^Department of Clinical Laboratory, Peking Union Medical College Hospital, Peking Union Medical College, Chinese Academy of Medical Sciences, Beijing, China; ^2^Graduate School, Peking Union Medical College, Chinese Academy of Medical Sciences, Beijing, China; ^3^Beijing Key Laboratory for Mechanisms Research and Precision Diagnosis of Invasive Fungal Diseases, Beijing, China; ^4^Charles Sturt University, Leeds Parade, Orange, NSW, Australia; ^5^Department of Clinical Laboratory, Peking University First Hospital, Beijing, China; ^6^Becton Dickinson Medical Devices Company, Shanghai, China; ^7^Centre for Infectious Diseases and Microbiology Laboratory Services, Westmead Hospital, Westmead, NSW, Australia; ^8^Department of General Surgery, Peking Union Medical College Hospital, Chinese Academy of Medical Sciences, Beijing, China

**Keywords:** blood culture, bloodstream infection, cost-effectively, direct identification, MALDI-TOF mass-spectrometry

## Abstract

**Background:** Bloodstream infection is a major cause of morbidity and mortality in hospitalized patients worldwide. Delays in the identification of microorganisms often leads to a poor prognosis. The application of matrix-assisted laser desorption/ionization time-of-flight mass spectrometry (MALDI-TOF MS) directly to blood culture (BC) broth can potentially identify bloodstream infections earlier, and facilitate timely management.

**Methods:** We developed an “in-house” (IH) protocol for direct MALDI-TOF MS based identification of organisms in positive BCs. The IH protocol was initially evaluated and improved with spiked BC samples, and its performance was compared with the commercial Sepsityper™ kit using both traditional and modified cut-off values. We then studied in parallel the performance of the IH protocol and the colony MS identifications in positive clinical BC samples using only modified cut-off values. All discrepancies were investigated by “gold standard” of gene sequencing.

**Results:** In 54 spiked BC samples, the IH method showed comparable results with Sepsityper™ after applying modified cut-off values. Specifically, accurate species and genus level identification was achieved in 88.7 and 3.9% of all the clinical monomicrobial BCs (284/301, 94.4%), respectively. The IH protocol exhibited superior performance for Gram negative bacteria than for Gram positive bacteria (92.8 vs. 82.4%). For anaerobes and yeasts, accurate species identification was achieved in 80.0 and 90.0% of the cases, respectively. For polymicrobial cultures (17/301, 5.6%), MALDI-TOF MS correctly identified a single species present in all the polymicrobial BCs under the Standard mode, while using the MIXED method, two species were correctly identified in 52.9% of the samples. Comparisons based on BC bottle type, showed that the BACTEC™ Lytic/10 Anaerobic/F culture vials performed the best.

**Conclusion:** Our study provides a novel and effective sample preparation method for MALDI-TOF MS direct identification of pathogens from positive BC vials, with a lower cost ($1.5 vs. $ 7) albeit a slightly more laborious extracting process (an extra 15 min) compared with Sepsityper™ kit.

## Introduction

Bloodstream infection is a major cause of morbidity and mortality in hospitalized patients worldwide, exhibiting a significant disease burden and negative economic impact (Klein et al., 2012). Each hour of delay in initiating an appropriate antimicrobial therapy has been associated with a 7.6% decrease in survival for a septic patient who remains untreated or receives inappropriate antimicrobial therapy within the first 24 h (Seifert, 2009). Blood culture (BC) remains the gold standard for the diagnosis of bloodstream infections, and relies on subsequent conventional techniques, including Gram staining, sub-culture followed by biochemical tests, or an automatic preformed enzyme assay, for identification and antimicrobial susceptibility testing of the pathogens. The whole procedure takes on average 12–48 h to complete (Caspar et al., 2017). Although several strategies have been employed to shorten the identification process (Peters et al., 2004, 2006; Forrest et al., 2008; Cattoir et al., 2010; Jukes et al., 2010), no satisfactory method is available for routine clinical use (Ferroni et al., 2010). Thus there is a continuous search for a simple, rapid, broad-spectrum, and cost-effective system for the direct identification of BC pathogens.

More recently, MALDI-TOF MS has revolutionized the identification of pathogens in clinical microbiology (Seng et al., 2009). In an attempt to assess the use of MALDI-TOF MS for the direct identification of pathogens in BCs, the Bruker company has launched a commercial kit, the Sepsityper™ (Bruker Daltonics, Bremen, Germany), to facilitate the quick pre-processing of positive BCs prior to spectrometric analysis (Nonnemann et al., 2013). Subsequently, various protocols based on stepwise centrifugation, washing, filtration and protein extraction, have been proposed (Machen et al., 2014; Jakovljev and Bergh, 2015; Riederer et al., 2015). Contrary to the Sepsityper™ kit, these “in house” (H) protocols are much more cost-effective, but can be tedious and lack standardization, making it difficult to compare and generalize results (Dubourg and Raoult, 2016). Hence, a simple method with good balance of both cost and performance is urgently needed in many clinical microbiology laboratories.

In this study, we aimed at developing a simple, reliable, and accurate IH processing method for positive BCs in preparation for spectrometric analysis. We then compared its performance to the commercial Sepsityper™ kit, for direct identification of pathogens from positive BCs using MALDI-TOF MS (Bruker Daltonics, Bremen, Germany). The intention was to assess the possibility of fully integrating the new rapid method into the diagnostic routine of a microbiological laboratory.

## Materials and methods

The first stage of this study was the development of an in-house (IH) protocol (see below), for rapid direct identification of organisms in positive BCs using MALDI-TOF MS. This involved evaluation of the performance of the IH protocol vs. Sepsityper™ kit method, through inoculating various known bacterial and fungal species into BC bottles. In the second stage, we further tested positive clinical BCs using the IH protocol, and the results obtained by direct MALDI-TOF MS were compared with colony MS identifications.

### Step 1: development of an improved protocol

#### Blood culture processing

Fifty-four blood samples (9 mL each), each from a healthy volunteer, were artificially spiked with 1 mL suspension of commonly isolated microorganisms with a final concentration of 10^4^ CFU/mL. The organisms used were previously identified by partial 16S r*RNA* gene, *gyrB* gene and internal transcribed spacer (ITS) region, sequencing (Table 1). For these organisms, template DNA was prepared as described by Dubois et al. (2013). The16S rRNA and ITS genes were amplified for bacteria and yeasts, respectively (Seng et al., 2009; Zhang et al., 2014). Purified PCR products and sequencing primers were mixed and sent to Ruibiotech (Beijing, China) for sequencing. Species identification was performed by comparing the obtained sequences against those in the GenBank database using BLASTn (www.ncbi.nlm.nih.gov/blast). A sequence similarity of 99% was used as species identification “cut-off” value. For viridans group streptococcus (VGS), an internal fragment of the *gyrB* gene was amplified, and a sequence similarity of 96% was used as species identification standard, according to Galloway-Pena et al. (Galloway-Pena et al., 2014; Zhou et al., 2016).

**Table 1:** Organisms used in spiked samples.

**Spiked isolates**	**Numbers**	**Gene sequencing**
**GRAM-NEGATIVE**		
*Escherichia coli*	2	Partial 16S rRNA gene
*Klebsiella pneumoniae*	2	Partial 16S rRNA gene
*Enterobacter cloacae*	2	Partial 16S rRNA gene
*Citrobacter freundii*	2	Partial 16S rRNA gene
*Serratia marcescens*	2	Partial 16S rRNA gene
*Morganella morganii*	2	Partial 16S rRNA gene
*Proteus mirabilis*	2	Partial 16S rRNA gene
*Pseudomonas aeruginosa*	2	Partial 16S rRNA gene
*Acinetobacter baumannii*	2	Partial 16S rRNA gene
Total Gram-negative	18	
**GRAM-POSITIVE**		
*Staphylococcus aureus*	2	Partial 16S rRNA gene
*Staphylococcus epidermidis*	2	Partial 16S rRNA gene
*Staphylococcus saprophyticus*	2	Partial 16S rRNA gene
*Staphylococcus hominis*	2	Partial 16S rRNA gene
*Enterococcus faecalis*	2	Partial 16S rRNA gene
*Enterococcus faecium*	2	Partial 16S rRNA gene
*Streptococcus pneumoniae*	2	Partial 16S rRNA gene & *gyrB* gene
*Streptococcus salivarius*	2	Partial 16S rRNA gene & *gyrB* gene
*Streptococcus mitis*	1	Partial 16S rRNA gene & *gyrB* gene
*Streptococcus oralis*	1	Partial 16S rRNA gene & *gyrB* gene
*Streptococcus anginosus*	2	Partial 16S rRNA gene & *gyrB* gene
*Streptococcus pyogenes*	2	Partial 16S rRNA gene
*Streptococcus agalactiae*	2	Partial 16S rRNA gene
Total Gram-positive	24	
***CANDIDA***		
*Candida albicans*	2	Internal Transcribed Spacer Regions
*Candida glabrata*	2	Internal Transcribed Spacer Regions
*Candida tropicalis*	2	Internal Transcribed Spacer Regions
*Candida parapsilosis*	2	Internal Transcribed Spacer Regions
*Issatchenkia orientalis*	2	Internal Transcribed Spacer Regions
Total *Candida*	10	
**ANAEROBES**		
*Bacteroides fragilis*	2	Partial 16S rRNA gene
Total Anaerobes	2	
Overall	54	

Each organism was inoculated into 2 BC vials (BACTEC™ Plus Aerobic/F culture vial (PAV) and BACTEC™ Lytic/10 Anaerobic/F culture vial (LAV), Becton Dickinson). The BC bottles were incubated at 37°C in an automated BC machine (BACTEC™ FX400, Becton Dickinson) for 7 days until flagged positive. When the instrument indicated that a bottle was positive, Gram staining was performed and a drop was sub-cultured on agar plates, and incubated overnight at 37°C. In parallel with direct identification by MALDI-TOF MS (using two sample preparation method described below), bacteria grown on solid media were spotted onto the ground steel target plate for MALDI-TOF MS identification.

#### Sample extraction methods for direct MALDI-TOF MS analysis

**Figure 1:**
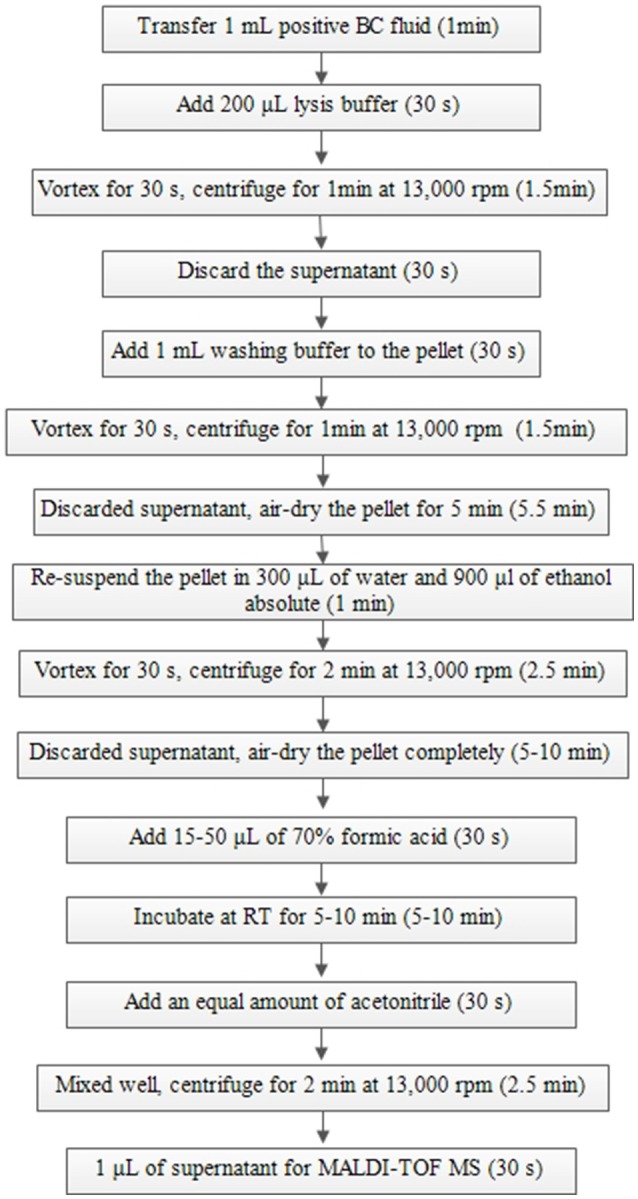
Sample preparation from positive BCs using a Sepsityper™ kit according to the manufacturer's instructions for direct MALDI-TOF MS.

**Figure 2:**
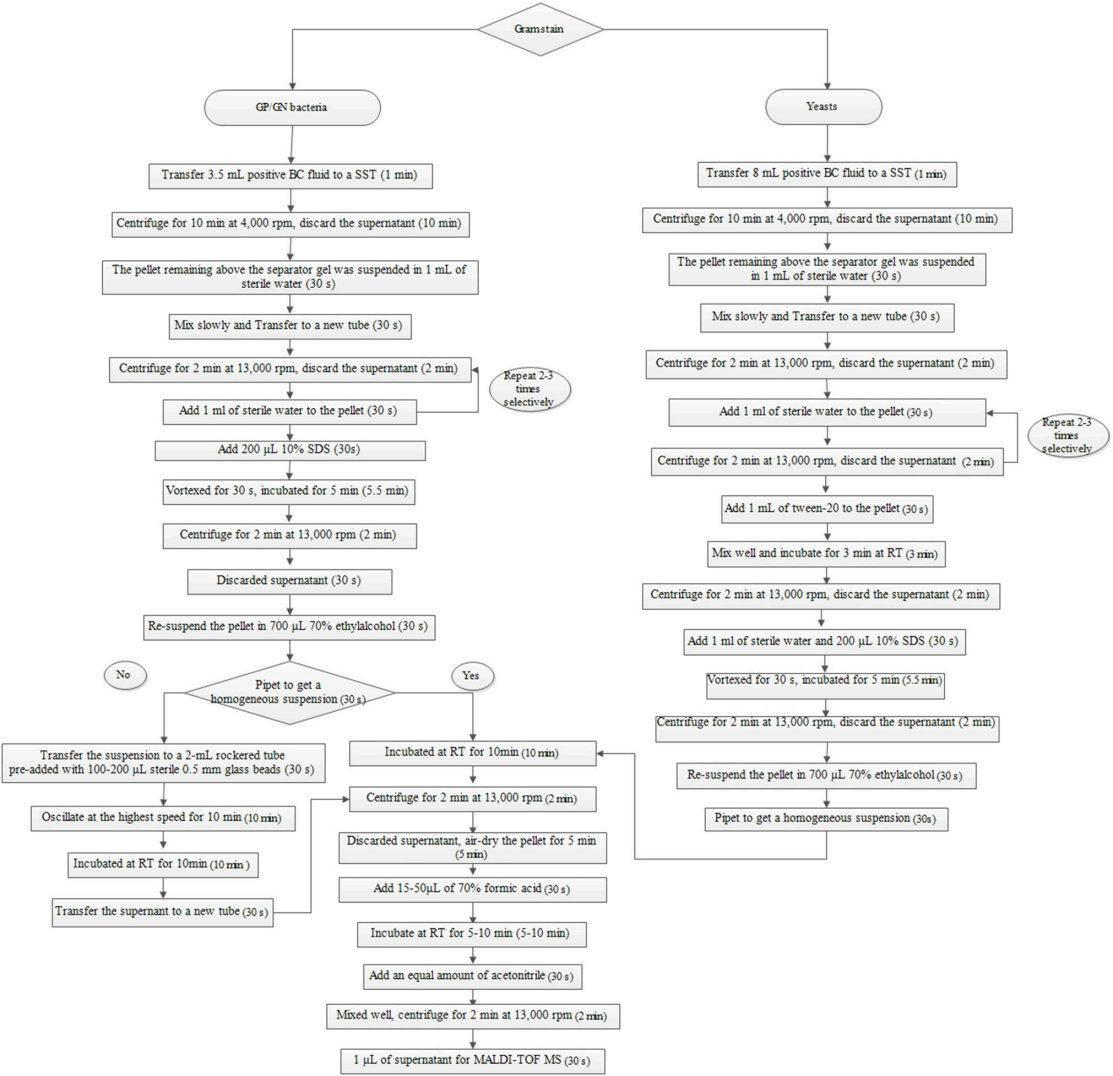
Sample preparation from positive BCs based on Gram staining using the IH protocol for direct MALDI-TOF MS. SST, serum separating tubes; SDS, sodium dodecyl sulfate; RT, room temperature.

The broth from each of the positive BCs (artificially spiked) was simultaneously treated with two preparatory (extraction) methods (the Sepsityper™ kit vs. the IH protocol) prior to direct MALDI-TOF MS analysis. Detailed information of the two protocols are shown in Figures 1, 2. Sample preparation for the Sepsityper™ kit was performed according to the manufacturer's instructions (Figure 1). For the IH protocol, based on the Gram stain results, bacteria and yeasts were processed differently (Figure 2). Notable differences in the present IH protocol compared to previous ones, include the use of glass beads in one of the stages for bacteria, and the addition of I mL tween-20 to the pellet before washing with sodium dodecyl sulfate (SDS), for yeasts (Figure 2) (Pulcrano et al., 2013; Paolucci et al., 2014; Rodriguez-Sanchez et al., 2014).

#### MALDI-TOF mass spectrometry

##### MALDI-TOF MS Settings

For direct MALDI-TOF MS analysis (using Sepsityper™ kit and IH protocol), duplicate 1 μL of extracted protein supernatant from the last centrifugation were applied to the polished steel target plate and once dried, were immediately overlaid with 1 μL α-cyano-4-hydroxycinnamic acid (HCCA) matrix (Bruker Daltonics, Bremen, Germany) solution before MALDI-TOF MS analysis. The final score used for interpretation for each sample was the higher one from two measurements. For MALDI-TOF MS analysis after BC subculture (standard method), a small portion of a single colony (after 24 or 48 h of incubation) was smeared onto the ground steel target plate using a wooden cocktail stick, and covered with 1 μL HCCA matrix solution immediately. Measurements were performed with the Bruker Biotyper MALDI-TOF MS system using FlexControl 3.3 and MALDI Biotyper V.3.3.1.2 software (Bruker Daltonics) as previously described (Zhou et al., 2016).

##### MALDI-TOF MS Analysis of Results

Two sets of criteria were used to analyze the identification results. Firstly, all results were evaluated according to the manufacturer's values recommended for identification after culture on solid media (traditional or standard cut-off values). Briefly, a score of <1.7 was interpreted as “no” identification, a score of 1.7–2.0 as identification to genus level, and a score of ≥2.0 as identification to species level. The results (identification scores) were then further evaluated according to modified cut-off values as per the manufacturer's recommendation for Sepsityper™ kit protocol. For bacteria, scores of 1.8 and 1.6 were considered as acceptable identification to species and genus levels (Nonnemann et al., 2013; Martinez et al., 2014), respectively. For yeasts, score values of ≥1.5 (provided the first two results were identical) and ≥1.4, were considered to be acceptable identification to the species and genus levels (Idelevich et al., 2014), respectively.

Based on these two criteria, the identification results were classified into the following categories: (i) correct species identification, (ii) correct genus identification, and (iii) misidentification or “no” identification.

### Step 2: validation in routine clinical workflow

#### Collection of blood cultures

In order to assess the utility of our IH protocol for routine clinical lab use, we analyzed its performance in clinical BCs. From June 1st to October 31st 2016, positive BCs for each bacteraemic episode in patients from Peking Union Medical College Hospital (PUMCH) were prospectively enrolled. Positive BCs from the same patient separated by more than a week were considered as different episodes. The positive BCs were Gram stained and sub-cultured for colony MALDI TOF MS identification. Furthermore, direct MALDI-TOF MS identification was performed but only using the IH protocol and the modified cut-off value interpretations. Discordant identification results between the direct MALDI-TOF MS result and the sub-cultured colony protocol, were resolved by molecular sequence (partial 16S rRNA gene, *gyrB* gene and ITS region) based identification.

#### Polymicrobial cultures

When microscopy of Gram stain on the clinical BCs revealed more than one morphology, or subculture on solid media showed polymicrobial growth, direct MALDI-TOF MS profiles were compared to the database using the MALDI Biotyper MSP identification MIXED Method in addition to the Standard Method, both of which can be chosen in the MALDI Biotyper V.3.3.1.2 software. Use of the Mixed Method is recommended by the manufacturer for identification of organisms in polymicrobial BCs (Bruker, Personal communication).

## Results

### Step 1: development of an improved protocol

#### Comparison of sample preparation using a sepsityper™ kit and the in-house (IH) protocol

A total of 54 spiked BC samples (44 bacteria and 10 yeasts) were tested as described above (Table 1), and both traditional (standard) and modified cut-off values were used in organism identification. Overall, 52 PAVs flagged positive except for two anaerobes, and 40 LAVs flagged positive except for ten *Candida* species, and four non-fermenting Gram negative (GN) bacilli for both method (Tables S1, S2).

#### Comparison of the two direct MALDI-TOF MS protocols using traditional cut-off values

Of all the flagged positive BCs, accurate species, genus, mis/no-identification, was achieved in 35/52 (67.4%), 14/52 (26.9%), 4/52 (7.7%) from PAVs, and 32/40 (80.0%), 7/40 (17.5%) and 1/40 (2.5%) from LAVs respectively, using the Sepsityper™ kit. This is in comparison to 29/52 (55.7%), 7/52 (30.8%), 7/52 (13.5%), and 25/40 (62.5%), 11/40 (27.5%) and 4/40 (10.0%) using the IH protocol, respectively (Table S1). Thus using the traditional cut-off values (recommended for MALDI-TOF identification of cultured isolates), Sepsityper™ kit showed an overall better identification than the IH protocol in both aerobic (67.4 vs. 55.7%, *p* = 0.3) and anaerobic (80.0% vs. 62.5%, *p* = 0.1) culture vials. Performance of the two protocols in relation to BC bottle type varied slightly in the different methods and species. LAVs had a higher detection rate of Gram positive (GP) bacteria for both methods, and for GN bacteria by Sepsityper™ kit. PAVs had higher detection rates of GN bacteria by the IH protocol (Table S1).

#### Comparison of the two direct MALDI-TOF MS protocols using the modified cut-off values

An overall increase in the identification rate was observed using the modified cut-off values irrespective of the method or culture vial deployed. Specifically, using the Sepsityper™ kit, accurate species identification increased from 67.4 to 82.7% for PAVs and from 80.0 to 95.0% for LAVs, compared to 55.7 to 84.6%, and 62.5 to 85.0%, using the IH protocol. Moreover, the IH method showed equal or even better performance in GP bacteria for both BC vials, and in GN bacteria for PAVs. For identification of GP bacteria in LAVs and *Candida* species, though not as good as the Sepsityper™ kit, the IH method achieved a significant accurate species identification increase from 37.5 to 75.0%, and from 40.0 to 80.0%, respectively (Table S2).

### Step 2: validation in clinical routine workflow

Based on the superior performance of the IH protocol in relation to Sepsityper™ kit, the performance of this protocol in clinical BCs was compared to the standard method (MALDI-TOF MS after subculture.) In five consecutive months, 301 positive BCs comprising 284 (94.4%) mono-microbial BCs (159 PAVs, 115 LAVs, and 10 BACTEC™ Myco/F Lytic Culture Vials (MLVs), and 17 (5.6%) polymicrobial BCs, were studied (Table 2).

**Table 2:** Direct organism identification by MALDI-TOF MS in clinical monobacterial blood cultures from patients.

**Organisms (Colony MALDI-TOF MS plus gene sequencing results)**	**No. of isolates**	**MALDI-TOF MS score**				**Isolates identified by MALDI-TOF MS at the species level**		**Isolates identified by MALDI-TOF MS at the genus level**		**Isolates mis/not identified by MALDI-TOF MS**	
		**<1.6**	**1.6–1.799**	**1.8–1.999**	**≥2.0**	**No**.	**Identity (%)**	**No**.	**Identity (%)**	**No**.	**Identity (%)**
***STAPHYLOCOCCUS*** **SPP**.											
*Staphylococcus aureus*	23	0	0	1	22	23	100.0	0	0.0	0	0.0
*Staphylococcus capitis*	1	0	0	1	0	1	100.0	0	0.0	0	0.0
*Staphylococcus epidermidis*	8	0	1	6	1	7	87.5	1	12.5	0	0.0
*Staphylococcus haemolyticus*	5	0	0	5	0	5	100.0	0	0.0	0	0.0
*Staphylococcus hominis*	7	0	1	1	5	6	85.7	1	14.3	0	0.0
*Staphylococcus pettenkoferi*	1	0	0	1	0	1	100.0	0	0.0	0	0.0
*Staphylococcus warneri*	1	0	0	0	1	1	100.0	0	0.0	0	0.0
Total *Staphylococcus* spp.	46	0	2	15	29	44	95.7	2	4.3	0	0.0
***STREPTOCOCCUS*** **SPP**.											
*Streptococcus constellatus*	5	0	1	3	1	4	80.0	1	20.0	0	0.0
*Streptococcus mitis*	2	1	0	0	1	0	0.0	0	0.0	2	100.0
*Streptococcus oralis*	13	0	0	10	3	11	84.6	0	0.0	2	15.4
*Streptococcus pneumoniae*	2	0	0	2	0	2	100.0	0	0.0	0	0.0
*Streptococcus pyogenes*	1	0	0	1	0	1	100.0	0	0.0	0	0.0
*Streptococcus sinensis*	4	3	0	1	0	1	25.0	0	0.0	3	75.0
*Streptococcus tigurinus*	2	0	0	0	2	0	0.0	0	0.0	2	100.0
Total *Streptococcus* spp.	29	4	1	17	7	19	65.5	1	3.5	9	31.0
***ENTEROCOCCUS*** **SPP**.											
*Enterococcus faecalis*	6	0	0	2	4	6	100.0	0	0.0	0	0.0
*Enterococcus faecium*	6	0	0	3	3	6	100.0	0	0.0	0	0.0
*Enterococcus gallinarum*	2	0	0	1	1	2	100.0	0	0.0	0	0.0
Total *Enterococcus* spp.	14	0	0	6	8	14	100.0	0	0.0	0	0.0
**OTHER GP COCCI**											
*Micrococcus luteus*	1	0	0	1	0	1	100.0	0	0.0	0	0.0
Total Other GP cocci	1	0	0	1	0	1	100.0	0	0.0	0	0.0
**GP RODS**											
*Corynebacterium afermentans*	2	1	1	0	0	0	0.0	1	50.0	1	50.0
*Corynebacterium amycolatum*	3	2	1	0	0	0	0.0	1	33.3	2	66.7
*Corynebacterium mucifaciens*	1	0	1	0	0	0	0.0	1	100.0	0	0.0
*Corynebacterium striatum*	4	0	0	1	3	4	100.0	0	0.0	0	0.0
*Corynebacterium tuberculostearicum*	1	0	0	1	0	1	100.0	0	0.0	0	0.0
*Listeria monocytogenes*	1	0	0	0	1	1	100.0	0	0.0	0	0.0
Total GP rods	12	3	3	2	4	6	50.0	3	25.0	3	25.0
Total GP bacteria	102	7	6	41	48	84	82.4	6	5.9	12	11.7
***ENTEROBACTERIACEAE***											
*Citrobacter freundii*	3	0	0	0	3	3	100.0	0	0.0	0	0.0
*Escherichia coli*	69	0	0	11	58	69	100.0	0	0.0	0	0.0
*Enterobacter aerogenes*	3	0	0	0	3	3	100.0	0	0.0	0	0.0
*Enterobacter cloacae*	9	0	0	2	7	9	100.0	0	0.0	0	0.0
*Klebsiella oxytoca*	2	0	0	0	2	2	100.0	0	0.0	0	0.0
*Klebsiella pneumoniae*	40	0	0	7	33	40	100.0	0	0.0	0	0.0
*Salmonella* spp.	3	0	0	1	2	0	0.0	3	100.0	0	0.0
Total *Enterobacteriaceae*	129	0	0	21	108	126	97.7	3	2.3	0	0.0
**NON-FERMENTING BACILLI**											
*Acinetobacter baumannii*	11	2	1	6	2	8	72.7	1	9.1	2	18.2
*Achromobacter xylosoxidans*	2	0	0	2	0	2	100.0	0	0.0	0	0.0
*Burkholderia multivorans*	1	0	0	1	0	1	100.0	0	0.0	0	0.0
*Moraxella osloensis*	1	0	0	1	0	1	100.0	0	0.0	0	0.0
*Ochrobactrum anthropi*	1	0	0	0	1	1	100.0	0	0.0	0	0.0
*Pseudomonas aeruginosa*	11	0	0	0	11	11	100.0	0	0.0	0	0.0
*Stenotrophomonas maltophilia*	4	0	1	2	1	3	75.0	1	25.0	0	0.0
*Sphingomonas pseudosanguinis*	1	0	0	0	1	1	100.0	0	0.0	0	0.0
Total Non-fermenting bacilli	32	2	2	12	16	28	87.4	2	6.3	2	6.3
**OTHER GN BACILLI**											
*Neisseria elongata*	3	3	0	0	0	0	0.0	0	0.0	3	100.0
*Vibrio cholerae*	2	2	0	0	0	0	0.0	0	0.0	2	100.0
Total Other GN bacilli	5	5	0	0	0	0	0.0	0	0.0	5	100.0
Total GN bacteria	166	7	2	33	124	154	92.8	5	3.0	7	4.2
**ANAEROBES**											
*Bacteroides fragilis*	1	0	0	0	1	1	100.0	0	0.0	0	0.0
*Clostridium aldenense*	1	0	0	0	1	1	100.0	0	0.0	0	0.0
*Peptoniphilus harei*	1	1	0	0	0	0	0.0	0	0.0	1	100.0
*Propionibacterium acnes*	2	0	0	0	2	2	100.0	0	0.0	0	0.0
Total Anaerobes	5	1	0	0	4	4	80.0	0	0.0	1	20.0
**YEAST**											
*Candida albicans*	1	0	1	0	0	1	100.0	0	0.0	0	0.0
*Candida glabrata*	1	1	0	0	0	1	100.0	0	0.0	0	0.0
*Candida parapsilosis*	4	0	1	3	0	4	100.0	0	0.0	0	0.0
*Candida tropicalis*	2	0	1	1	0	2	100.0	0	0.0	0	0.0
*Meyerozyma guilliermondii*	1	1	0	0	0	0	0.0	0	0.0	1	100.0
*Trichosporon asahii*	2	1	1	0	0	2	100.0	0	0.0	0	0.0
Total Yeast	11	3	4	4	0	10	90.9	0	0.0	1	9.1
Overall	284	18	12	78	176	252	88.7	11	3.9	21	7.4

*Scores Distribution: The MALDI-TOF MS reported scores of ≥2.0 in 176/284 (62.0%) isolates, scores of 1.8–1.999 in 78/284 (27.5%), scores of 1.6–1.799 in12/284 (4.2%) and scores <1.6 in 18/294 (6.3%) of the monomicrobial BCs*.

*Species Identification: (a) For GP bacteria, 44/46 (95.7%) of Staphylococcus, 19/29 (65.5%) of Streptococcus, 14/14 (100.0%) of Enterococcus, 1/1 (100.0%) of other GP cocci and 6/12 (50.0%) of GP rods were correctly identified to species level. (b) For GN bacteria, 126/129 (97.9%) of Enterobacteriaceae, 28/32 (87.4%) of non-fermenting bacilli, 0/5 (0.0%) for other GN bacilli were correctly identified to species level*.

#### Sequencing-based identification

Only samples with discrepant results between direct and colony MS identification, and all VGS, were subjected to gene sequencing. A total of 20 such cases, including three *Streptococcus sinensis*, two each of; *Acinetobacter baumannii, Corynebacterium amycolatum, Neisseria elongate, Streptococcus mitis, Streptococcus oralis, Streptococcus tigurinus, Vibrio cholera*, and one each of; *Corynebacterium afermentans, Peptoniphilus harei*, and *Meyerozyma guilliermondii*, were identified. In 15 (75%) of the cases, the molecular sequencing results were in agreement with colony MS results; one was accordant with direct MS result (*S. mitis*) and the remaining four discordant with either (one *S. sinensis*, one *S. mitis* and two *S. tigurinus*).

#### Direct identification of monomicrobial cultures using the IH protocol

Among the 284 monomicrobial BCs, 102 were GP, 166 GN, 5 anaerobes, and 11 yeasts, representing 27 genera and 51 species or groups (Table 2). Using the modified cut-off values, which is recommended for methods involving a prior preparation step, accurate species, genus, mis/no-identification (as compared to colony MALDI-TOF MS) was achieved in 252/284 (88.7%), 11/284 (3.9%), and 21/284 (7.4%) of all the monomicrobial BCs. The IH protocol (using the modified cut-off values) showed better performance with GN bacteria than for GP bacteria in general (species ID: 92.8 vs. 82.4%; *p* = 0.01). Variation in accurate species identification rate was also observed among different species or genera (Table 2). As for anaerobes and yeasts, correct species identification rate was achieved for 4/5 (80.0%) and 10/11 (90.0%) of the cases, respectively.

#### Direct identification of polymicrobial cultures using the IH protocol

Of the 17 polymicrobial BCs, direct MALDI-TOF MS correctly reported a single species present in all the polymicrobial BCs, with 16/17 (94.1%) correctly identified to the species level and 1/17 (5.9%) to the genus level, under the Standard mode. However, using the MIXED Method, which the manufacturer recommends for suspected mixed organisms, two species were identified in 9/17 (52.9%) of all the episodes, among which 4/17 (23.5%) were accordant with colony MS results, and 5/17 (29.4%) were partially correct. The remaining cases with only one species identified, presented similar results to the Standard Method (Table 3).

**Table 3:** Direct identification by MALDI-TOF MS in clinical blood cultures containing more than 2 organisms.

**No**.	**Gram stain results**	**Microorganism(s) (Colony MALDI-TOF MS plus gene sequencing results)**	**Identification using MALDI Biotyper MSP identification standard method**		**Identification using MALDI Biotyper MSP identification MIXED method**	
			**Species**	**Score**	**Species**	**Score**
1	GP	*Enterococcus faecalis*	*Enterococcus faecalis*	1.939	*Enterococcus faecalis*	1.939
		*Staphylococcus chromogenes*				
		*Staphylococcus hyicus*				
2	GP	*Enterococcus faecalis*	*Enterococcus faecalis*	2.067	*Enterococcus faecalis*	2.067
		*Staphylococcus hyicus*				
3	GP	*Staphylococcus aureus*	*Staphylococcus aureus*	2.034	*Enterococcus faecalis*+ *Staphylococcus aureus*	2.435
		*Enterococcus faecalis*				
		*Staphylococcus hyicus*				
4	GP	*Staphylococcus aureus*	*Enterococcus faecalis*	1.938	*Enterococcus faecalis*	1.938
		*Enterococcus faecalis*				
5	GP	*Staphylococcus hyicus*	*Enterococcus faecalis*	1.888	*Enterococcus faecalis*	1.888
		*Staphylococcus aureus*				
		*Enterococcus faecalis*				
6	GP	*Corynebacterium striatum*	*Enterococcus faecalis*	2.15	*Enterococcus faecalis*	2.15
		*Enterococcus faecalis*				
		*Staphylococcus epidermidis*				
7	GP	*Staphylococcus hominis*	*Staphylococcus epidermidis*	1.727	*Staphylococcus epidermidis*	1.727
		*Staphylococcus haemolyticus*				
		*Staphylococcus epidermidis*				
8	GN	*Escherichia coli*	*Escherichia coli*	2.143	*Escherichia coli* +*Klebsiella pneumoniae*	2.587
		*Klebsiella pneumoniae*				
9	GN	*Escherichia coli*	*Escherichia coli*	2.222	*Escherichia coli* +*Klebsiella pneumoniae*	2.604
		*Klebsiella pneumoniae*				
10	GP, GN	*Pseudomonas aeruginosa*	*Escherichia coli*	2.296	*Escherichia coli* +*Raoultella ornithinolytica*	2.563
		*Escherichia coli*				
		*Streptococcus gallolyticus*				
11	GP, GN	*Klebsiella pneumoniae*	*Streptococcus gallolyticus*	2.114	*Streptococcus gallolyticus* + *Streptococcus equinus*	2.214
		*Streptococcus gallolyticus*				
12	GP	*Staphylococcus cohnii*	*Staphylococcus cohnii*	2.045	*Staphylococcus cohnii*	2.045
		*Staphylococcus epidermidis*				
13	GP	*Staphylococcus epidermidis*	*Staphylococcus epidermidis*	2.009	*Staphylococcus epidermidis* + *Staphylococcus pasteuri*	2.226
		*Micrococcus luteus*				
14	GP	*Staphylococcus epidermidis*	*Staphylococcus epidermidis*	1.842	*Staphylococcus epidermidis* + *Staphylococcus aureus*	2.132
		*Micrococcus luteus*				
15	GP, GN	*Enterococcus faecium*	*Escherichia coli*	2.138	*Escherichia coli* + *Enterococcus faecium*	2.477
		*Escherichia coli*				
16	GP, GN	*Enterococcus faecium*	*Escherichia coli*	2.407	*Escherichia coli* +*Citrobacter amalonaticus*	2.563
		*Enterococcus faecalis*				
		*Escherichia coli*				
17	GP	*Acinetobacter baumannii*	*Enterococcus faecium*	1.906	*Enterococcus faecium*	1.906
		*Enterococcus faecium*				

*Of the 17 polymicrobial BC broths, ten presented with two different organisms and seven with three different organisms*.

#### Performance of direct MALDI-TOF MS in different BC vials using the IH protocol

Overall, accurate species identification was achieved in 87.4% (139/159) of PAVs, 92.1% (106/115) of LAVs, and 80.0% (8/10) of MLVs (Table S3). The LAVs provided the highest accurate identification rates for GP and GN bacteria, and overall. Regardless of the BC vials used, the identification accuracy for GN bacteria was generally higher than for GP bacteria (PAVs 82.5% (GP) vs. 90.3% (GN); LAVs 85.7% vs. 97.0%; MLVs 66.7% vs. 80.0%). Similar to the spiked BCs, none of the non-fermenting bacilli or yeasts flagged positive in LAVs. Noteworthy, only 2/11 (18.2%) of the yeasts flagged positive in MLVs, while the remaining 9/11 (81.8%) all flagged positive in PAVs.

## Discussion

In this study, we evaluated the performance of two extraction methods (Sepsityper™ kit vs. IH method) for MALDI-TOF MS direct identification of pathogens from blood culture (BC) vials. Using the modified cut-off values which is recommended for direct BC testing protocols (such as Sepsityper™ kit), a significant increase of about 15 and 25% was achieved in the accurate species identification rate in the MALDI Sepsityper™ kit and the IH method, respectively (Tables S1, S2), which agrees with previous studies (Gorton et al., 2014). Furthermore, the IH method showed similar or even better performance in comparison to the Sepsityper™ kit, except for Gram positive (GP) bacteria in LAVs.

A major difference between the current IH protocol and previous ones (Machen et al., 2014; Jakovljev and Bergh, 2015), is the addition of sterile 0.5 mm glass beads to get a homogeneous suspension. In most cases, we discovered that this extra step enhanced the identification of GP bacteria, possibly due to the thick peptidoglycan layer of the cell wall (Klein et al., 2012). However, some mucous GN bacteria such as *Acinetobacter baumannii, Klebsiella* spp, also benefited from this treatment with beads. Save for the special processing procedure in the current study, our findings are in agreement with previous studies confirming that direct MALDI-TOF accurately identifies GN bacteria than GP (species ID: 92.8 vs. 82.4%, *p* = 0.01) (Nonnemann et al., 2013).

Identification problems for GP bacteria were confined to *Streptococcus* (species ID: 65.5%) and GP rods (species ID: 50.0%). Specifically, five (1 *S. mitis*, 2 *S. oralis*, 2 *S. tigurinus*) out of nine incorrect identifications were VGS misidentified as *S. pneumoniae*, while the remaining four (1 *S. mitis*, 3 *S. sinensis*) were unreliable results. This is not surprising as the high similarity in the protein profiles generated by *S. pneumoniae* and VGS (specifically the mitis group), is well known and acknowledged by the manufacturer (Zhou et al., 2016). Thus, it is advisable to report these isolates as *S. pneumoniae*/*S. mitis* group, pending additional identification tests. For GP rods, 12 cases (including 10 *Corynebacterium* spp. and 2 *Listeria monocytogenes*) were included in this study, of which only half were correctly identified to the species level. This is understandable considering that MALDI-TOF MS is reported to have difficulties in identifying GP rods even from plate cultures (Caspar et al., 2017). In contrast to previously encountered problems in the identification of *Staphylococcus* spp. (Rodriguez-Sanchez et al., 2014), the present IH protocol performed excellently, with no *S. aureus* misidentified as other coagulase negative Staphylococci and vice versa.

The IH protocol performed very well in the identification of GN bacteria, with almost all the *Enterobacteriaceae* correctly identified (and with excellent scores), except for the genus *Salmonella*. Non-discrimination of the *Salmonella* serotypes is a well-known limitation of MALDI-TOF MS identification (Neville et al., 2011). Problems regarding lower score values were observed in sporadic cases of *Acinetobacter baumannii, Stenotrophomonas maltophilia, Neisseria elongate* and *Vibrio cholera*, which might be theoretically explained by differences in the bacterial load, cell wall composition, background signals, as well as insufficient proteomic profiles in the database (Ferroni et al., 2010). Previous studies have shown that leucocytes and plasma in the blood can affect the size of the bacterial cell pellet after centrifugation which interferes with the identification confidence level. Furthermore, additional peaks may be caused by blood components, which could confuse the system and hamper the detection of the true microbial peaks or worse still, make them undetectable (Klein et al., 2012).

The newly designed IH protocol also performed better in the identification of anaerobes (80%) and yeasts (90%), than previously reported (Paolucci et al., 2014; Rodriguez-Sanchez et al., 2014), albeit few tested isolates. Several factors may contribute to this. Firstly, in our protocol, the gel-containing tubes were used to separate blood component from targeted fungus and tween-20 was added to the pellet for a 3-min incubation before SDS processing, which hasn't been described before. Secondly, a high volume of culture broth (8 mL) was adjusted for microbial enrichment according to preliminary experimental results. We tried 3.5 ml for positive yeasts bottles and the results were unsatisfactory. We adjusted the blood volume for yeasts to 8 ml for microbial enrichment, and the results improved. We chose 3.5 ml and 8 ml as the BD Vacutainer® SST™ only provides for these two specifications. Apart from limiting the potential loss of yeasts during extraction as mentioned above, Marinach-Patrice et al. used a statistical approach for spectral analysis in the 5,000–7,400 Da mass range to allow for better discrimination among 6 yeast species (Gorton et al., 2014). However, although our IH protocol performed well in the identification of yeasts, it was only validated in simulated BCs and was limited to only 6 *Candida* species. This study highlights that sample processing is a critical step for correct identification of yeasts by direct MALDI-TOF.

Another interesting feature of the current study is the use of MALDI Biotyper MSP MIXED Method to identify organisms in polymicrobial infections. In most laboratories, Gram staining positive BCs remains a cornerstone in diagnostics until subcultures are available, but lacks high specificity. In this study, six of the 17 polymicrobial BCs contained both GP and GN bacteria, but only five showed concordant Gram staining results. In the majority of the cases, the most abundant organism detected by Gram staining was the one identified by MALDI-TOF–MS using the Standard Method (Table 3). However, by applying the MIXED Method, two species were identified in 52.9% (9/17) of the episodes, among which 23.5% (4/17) were accordant with colony MS results, and 29.4% (5/17) were partially correct (one species accurately identified and another potential contaminative or closely related species). Although the results were unsatisfactory, this has not been achieved before, and may provide some preliminary information for management of patients with more than one organism in the BC. Recently, Ferroni et al. showed that by using GP- and GN-specific databases based on the BC Gram stain result could enhance the identification of both GP and GN bacteria effectively (Ferroni et al., 2010). However, for this to be a reality, specific Gram stain-based databases are needed, but may not be efficient for polymicrobial infections within the same Gram stain class.

Our study also revealed that irrespective of the type of BC bottle used, the identification accuracy of GN using the MALDI-TOF assay, is always better than that of GP bacteria. Studies in the performance of different BC bottles in the identification of different organisms, have yielded contrasting results. In this study, LAVs showed a significantly higher detection rate than the other two, which is in agreement to a previous study (Almuhayawi et al., 2015). On the contrary, Gray et al. reported that PAV culture vials had a higher median score and significantly improved spectra quality and identification rate in direct MALDI-OF MS compared with the LAVs (Moussaoui et al., 2010). The disparity in the study findings might be due to differences in the bottle composition or the underlying extraction protocols.

In a laboratory setting, the IH method is far more cost effective than the Sepsityper™ kit, costing about $1.5 per sample compared to $7 for the Sepsityper™ kit (Caspar et al., 2017). Considering the total number of positive BCs (2756) processed last year in the study hospital, replacement of the Sepsityper™ kit with the IH method would save the hospital approximately $15,000 per year.

Our study has some limitations. First, there is a possible selection bias as all data was from a single center with imbalance in group/species distribution. Second, we did not compare the performance of the IH protocol vs. Sepsityper™ kit in clinical BCs, which would be considered a weakness of the study. Further evaluation using the Sepsityper™ protocol needs to done for clinical samples. However, we compared the two protocols using simulated BCs and demonstrated the superior performance of the IH protocol. Therefore it made sense to compare the performance of the IH protocol to the routinely used MALDI-TO Ms colony method. Third, our protocol is somewhat more laborious and time-consuming compared to the Sepsityper™ kit and other IH protocols (Jakovljev and Bergh, 2015; Caspar et al., 2017), with an extra 10–17 min required on the turnaround time of about 35–50 min. However, taking into consideration the cost, and the increased accuracy achieved by IH protocol, we think the extra processing time is worth it. Furthermore, higher volumes of blood are used in the processing (which possibly increases the identification accuracy), but the results are more accurate and reliable, especially for *Staphylococcus* and yeasts. Fourth, for polymicrobial infections, though the MIXED Method was used, the results were barely satisfactory. And finally, our protocol is reliant on the working pattern of a particular laboratory. Indeed, our laboratory works 24 h with a night shift rotation, which allows a rapid processing of positive BCs.

## Summary

The quick identification of organisms in BCs even without antimicrobial susceptibilities could help clinicians make patient-tailored treatment more accurately, reducing the risk of potential development of resistance and possible side effects due to empirical broad-spectrum antibiotic therapy. In this respect, our study provides a novel sample preparation method for direct identification of pathogens from positive BCs with easy performance and low additional costs compared with the Sepsityper™ kit. The protocol exhibited an overall equal or even better performance than Sepsityper™ kit especially for yeasts, and showed better performance for GN bacteria than GP bacteria, and for LAVs than PAVs.

## Ethics statement

This study was carried out in accordance with the recommendations of Peking Union Medical College Hospital, Chinese Academy of Medical Sciences, Peking Medical College Hospital ethics committee with written informed consent from all subjects. All subjects gave written informed consent in accordance with the Declaration of Helsinki. The protocol was approved by Peking Union Medical College Hospital, Chinese Academy of Medical Sciences, Peking Medical College Hospital ethics committee.

## Author contributions

MZ, QY, and YX conceived and designed the experiments, performed the experiments, analyzed the data, and wrote the paper. TK and FK revised the paper critically for important intellectual content. LS, RZ, MX, CL, SY, and YZ read and approved the final version of the manuscript.

### Conflict of interest statement

The authors declare that the research was conducted in the absence of any commercial or financial relationships that could be construed as a potential conflict of interest. The reviewer AV and handling Editor declared their shared affiliation.

## Abbreviations

MALDI-TOF MS: matrix-assisted laser desorption ionization-time of flight mass spectrometry
VGS: viridans group streptococci
CoNS: coagulase negative staphylococcus
PUMCH: Peking Union Medical College Hospital
IH: in-house
BC: blood culture.
