# Supplementary material for: An Improved In-house MALDI-TOF MS Protocol for Direct Cost-Effective Identification of Pathogens from Blood Cultures
Source: Front Microbiol. 2017 Sep 28;8:1824. doi: 10.3389/fmicb.2017.01824 (PMC5625089; doi:10.3389/fmicb.2017.01824)
Supplement: Supplementary file 1 [file Table1.DOCX]

**An improved in-house MALDI-TOF MS protocol for direct cost-effective identification of pathogens from blood cultures**

**Menglan Zhou^1,2,3^, Qiwen Yang^1,3*^, Timothy Kudinha^4^, Liying Sun^5^, Rui Zhang^6^, Chang Liu^1,2,3^, Shuying Yu^1,2,3^, Meng Xiao^1,3^, Fanrong Kong^7^, Yupei Zhao^8^, Ying-Chun Xu^1,3^****^*^**

***Correspondence: Qiwen Yang: yangqiwen81@163.com, YingChun Xu: xycpumch@139.com**

**Supplementary Table S1. Identification results of spiked bottles by both methods using traditional cut-off values.**

| Identification |  | Sepsityper™ kit method | | | | | | In-house method | | | | | |
| --- | --- | --- | --- | --- | --- | --- | --- | --- | --- | --- | --- | --- | --- |
|  | No of isolates | BD BACTEC^TM^ Plus Aerobic/F | | | BD BACTEC^TM^ Lytic/10 Anaerobic/F | | | BD BACTEC^TM^ Plus Aerobic/F | | | BD BACTEC^TM^ Lytic/10 Anaerobic/F | | |
|  |  | Species | Genus | Mis/No ID | Species | Genus | Mis/No ID | Species | Genus | Mis/No ID | Species | Genus | Mis/No ID |
| Gram-negative |  |  |  |  |  |  |  |  |  |  |  |  |  |
| *Escherichia coli* | 2 | 100.0% | 0.0% | 0.0% | 100.0% | 0.0% | 0.0% | 100.0% | 0.0% | 0.0% | 100.0% | 0.0% | 0.0% |
| *Klebsiella pneumoniae* | 2 | 100.0% | 0.0% | 0.0% | 100.0% | 0.0% | 0.0% | 50.0% | 50.0% | 0.0% | 100.0% | 0.0% | 0.0% |
| *Enterobacter cloacae* | 2 | 100.0% | 0.0% | 0.0% | 100.0% | 0.0% | 0.0% | 100.0% | 100.0% | 0.0% | 100.0% | 0.0% | 0.0% |
| *Citrobacterfreundii* | 2 | 100.0% | 0.0% | 0.0% | 100.0% | 0.0% | 0.0% | 100.0% | 0.0% | 0.0% | 100.0% | 0.0% | 0.0% |
| *Serratiamarcescens* | 2 | 100.0% | 0.0% | 0.0% | 100.0% | 0.0% | 0.0% | 50.0% | 50.0% | 0.0% | 100.0% | 0.0% | 0.0% |
| *Morganellamorganii* | 2 | 100.0% | 0.0% | 0.0% | 100.0% | 0.0% | 0.0% | 100.0% | 0.0% | 0.0% | 100.0% | 0.0% | 0.0% |
| *Proteus mirabilis* | 2 | 100.0% | 0.0% | 0.0% | 100.0% | 0.0% | 0.0% | 100.0% | 0.0% | 0.0% | 100.0% | 0.0% | 0.0% |
| *Pseudomonas aeruginosa* | 2 | 100.0% | 0.0% | 0.0% | NFP | NFP | NFP | 100.0% | 0.0% | 0.0% | NFP | NFP | NFP |
| *Acinetobacter baumannii* | 2 | 50.0% | 50.0% | 0.0% | NFP | NFP | NFP | 50.0% | 50.0% | 0.0% | NFP | NFP | NFP |
| **Total Gram-negative** | **18** | **94.4%** | **5.6%** | **0.0%** | **100.0%** | **0.0%** | **0.0%** | **83.3%** | **16.7%** | **0.0%** | **100.0%** | **0.0%** | **0.0%** |
| Gram-positive |  |  |  |  |  |  |  |  |  |  |  |  |  |
| *Staphylococcus aureus* | 2 | 50.0% | 50.0% | 0.0% | 100.0% | 0.0% | 0.0% | 50.0% | 0.0% | 50.0% | 50.0% | 50.0% | 0.0% |
| *Staphylococcus epidermidis* | 2 | 0.0% | 100.0% | 0.0% | 50.0% | 50.0% | 0.0% | 100.0% | 0.0% | 0.0% | 100.0% | 0.0% | 0.0% |
| *Staphylococcus saprophyticus* | 2 | 0.0% | 100.0% | 0.0% | 0.0% | 100.0% | 0.0% | 0.0% | 100.0% | 0.0% | 0.0% | 100.0% | 0.0% |
| *Staphylococcus hominis* | 2 | 100.0% | 0.0% | 0.0% | 100.0% | 0.0% | 0.0% | 50.0% | 50.0% | 0.0% | 0.0% | 100.0% | 0.0% |
| *Enterococcus faecalis* | 2 | 100.0% | 0.0% | 0.0% | 50.0% | 50.0% | 0.0% | 0.0% | 100.0% | 0.0% | 50.0% | 50.0% | 0.0% |
| *Enteroccoccus faecium* | 2 | 100.0% | 0.0% | 0.0% | 100.0% | 0.0% | 0.0% | 100.0% | 0.0% | 0.0% | 50.0% | 50.0% | 0.0% |
| *Streptococcus pneumoniae* | 2 | 0.0% | 0.0% | 100.0% | 0.0% | 50.0% | 50.0% | 0.0% | 100.0% | 0.0% | 0.0% | 50.0% | 50.0% |
| *Streptococcus salivarius* | 2 | 0.0% | 100.0% | 0.0% | 100.0% | 0.0% | 0.0% | 0.0% | 100.0% | 0.0% | 50.0% | 0.0% | 50.0% |
| *Streptococcus mitis* | 1 | 0.0% | 0.0% | 100.0% | 0.0% | 100.0% | 0.0% | 0.0% | 0.0% | 100.0% | 0.0% | 0.0% | 100.0% |
| *Streptococcus oralis* | 1 | 0.0% | 100.0% | 0.0% | 0.0% | 100.0% | 0.0% | 0.0% | 0.0% | 100.0% | 0.0% | 0.0% | 100.0% |
| *Streptococcus anginosus* | 2 | 50.0% | 50.0% | 0.0% | 100.0% | 0.0% | 0.0% | 50.0% | 0.0% | 50.0% | 0.0% | 100.0% | 0.0% |
| *streptococcus pyogenes* | 2 | 100.0% | 0.0% | 0.0% | 100.0% | 0.0% | 0.0% | 50.0% | 50.0% | 0.0% | 50.0% | 50.0% | 0.0% |
| *streptococcus agalactiae* | 2 | 50.0% | 50.0% | 0.0% | 100.0% | 0.0% | 0.0% | 100.0% | 0.0% | 0.0% | 100.0% | 0.0% | 0.0% |
| **Total Gram-positive** | **24** | **45.8%** | **41.7%** | **12.5%** | **66.7%** | **29.2%** | **4.1%** | **45.8%** | **41.7%** | **12.5%** | **37.5%** | **50.0%** | **12.5%** |
| *Candida* |  |  |  |  |  |  |  |  |  |  |  |  |  |
| *Candida albicans* | 2 | 100.0% | 0.0% | 0.0% | NFP | NFP | NFP | 50.0% | 0.0% | 50.0% | NFP | NFP | NFP |
| *Candida glabrata* | 2 | 100.0% | 0.0% | 0.0% | NFP | NFP | NFP | 100.0% | 0.0% | 0.0% | NFP | NFP | NFP |
| *Candida tropicalis* | 2 | 50.0% | 50.0% | 0.0% | NFP | NFP | NFP | 0.0% | 50.0% | 50.0% | NFP | NFP | NFP |
| *Candida parapsilosis* | 2 | 0.0% | 100.0% | 0.0% | NFP | NFP | NFP | 50.0% | 50.0% | 0.0% | NFP | NFP | NFP |
| *Issatchenkiaorientalis* | 2 | 100.0% | 0.0% | 0.0% | NFP | NFP | NFP | 0.0% | 50.0% | 50.0% | NFP | NFP | NFP |
| **Total *Candida*** | **10** | **70.0%** | **30.0%** | **0.0%** | **NFP** | **NFP** | **NFP** | **40.0%** | **30.0%** | **30.0%** | **NFP** | **NFP** | **NFP** |
| Anaerobes |  |  |  |  |  |  |  |  |  |  |  |  |  |
| *Bacteroidesfragilis* | 2 | NFP | NFP | NFP | 100.0% | 0.0% | 0.0% | NFP | NFP | NFP | 100.0% | 0.0% | 0.0% |
| **Total Anaerobes** | **2** | **NFP** | **NFP** | **NFP** | **100.0%** | **0.0%** | **0.0%** | **NFP** | **NFP** | **NFP** | **100.0%** | **0.0%** | **0.0%** |
| **Overall** | **54** | **67.4%** | **26.9%** | **7.7%** | **80.0%** | **17.5%** | **2.5%** | **55.7%** | **30.8%** | **13.5%** | **62.5%** | **27.5%** | **10.0%** |

NFP: Not Flagged Positive

**Supplementary Table S2. Identification results of spiked bottles by both methods using modified cut-off values.**

| Identification |  | Sepsityper™ kit method | | |  |  |  |  |  |  | In-house method | |  |  |
| --- | --- | --- | --- | --- | --- | --- | --- | --- | --- | --- | --- | --- | --- | --- |
|  | No of isolates | BD BACTEC^TM^ Plus Aerobic/F | | |  | BD BACTEC^TM^ Lytic/10 Anaerobic/F | | | BD BACTEC^TM^ Plus Aerobic/F | | | BD BACTEC^TM^ Lytic/10 Anaerobic/F | | |
|  |  | Species | Genus | Mis/No ID | Species | Genus |  | Mis/No ID | Species | Genus | Mis/No ID | Species | Genus | Mis/No ID |
| Gram-negative |  |  |  |  |  |  |  |  |  |  |  |  |  |  |
| *Escherichia coli* | 2 | 100.0% | 0.0% | 0.0% | 100.0% | 0.0% |  | 0.0% | 100.0% | 0.0% | 0.0% | 100.0% | 0.0% | 0.0% |
| *Klebsiella pneumoniae* | 2 | 100.0% | 0.0% | 0.0% | 100.0% | 0.0% |  | 0.0% | 50.0% | 50.0% | 0.0% | 100.0% | 0.0% | 0.0% |
| *Enterobacter cloacae* | 2 | 100.0% | 0.0% | 0.0% | 100.0% | 0.0% |  | 0.0% | 100.0% | 0.0% | 0.0% | 100.0% | 0.0% | 0.0% |
| *Citrobacterfreundii* | 2 | 100.0% | 0.0% | 0.0% | 100.0% | 0.0% |  | 0.0% | 100.0% | 0.0% | 0.0% | 100.0% | 0.0% | 0.0% |
| *Serratiamarcescens* | 2 | 100.0% | 0.0% | 0.0% | 100.0% | 0.0% |  | 0.0% | 100.0% | 0.0% | 0.0% | 100.0% | 0.0% | 0.0% |
| *Morganellamorganii* | 2 | 100.0% | 0.0% | 0.0% | 100.0% | 0.0% |  | 0.0% | 100.0% | 0.0% | 0.0% | 100.0% | 0.0% | 0.0% |
| *Proteus mirabilis* | 2 | 100.0% | 0.0% | 0.0% | 100.0% | 0.0% |  | 0.0% | 100.0% | 0.0% | 0.0% | 100.0% | 0.0% | 0.0% |
| *Pseudomonas aeruginosa* | 2 | 100.0% | 0.0% | 0.0% | NFP | NFP |  | NFP | 100.0% | 0.0% | 0.0% | NFP | NFP | NFP |
| *Acinetobacter baumannii* | 2 | 50.0% | 0.0% | 50.0% | NFP | NFP |  | NFP | 100.0% | 0.0% | 0.0% | NFP | NFP | NFP |
| **Total Gram-negative** | **18** | **94.4%** | **5.6%** | **0.0%** | **100.0%** | **0.0%** |  | **0.0%** | **94.4%** | **5.6%** | **0.0%** | **100.0%** | **0.0%** | **0.0%** |
| Gram-positive |  |  |  |  |  |  |  |  |  |  |  |  |  |  |
| *Staphylococcus aureus* | 2 | 50.0% | 50.0% | 0.0% | 100.0% | 0.0% |  | 0.0% | 50.0% | 50.0% | 0.0% | 100.0% | 0.0% | 0.0% |
| *Staphylococcus epidermidis* | 2 | 50.0% | 50.0% | 0.0% | 100.0% | 0.0% |  | 0.0% | 100.0% | 0.0% | 0.0% | 100.0% | 0.0% | 0.0% |
| *Staphylococcus saprophyticus* | 2 | 100.0% | 0.0% | 0.0% | 100.0% | 0.0% |  | 0.0% | 100.0% | 0.0% | 0.0% | 50.0% | 50.0% | 0.0% |
| *Staphylococcus hominis* | 2 | 100.0% | 0.0% | 0.0% | 100.0% | 0.0% |  | 0.0% | 100.0% | 0.0% | 0.0% | 100.0% | 0.0% | 0.0% |
| *Enterococcus faecalis* | 2 | 100.0% | 0.0% | 0.0% | 50.0% | 50.0% |  | 0.0% | 100.0% | 0.0% | 0.0% | 100.0% | 0.0% | 0.0% |
| *Enteroccoccus faecium* | 2 | 100.0% | 0.0% | 0.0% | 100.0% | 0.0% |  | 0.0% | 100.0% | 0.0% | 0.0% | 100.0% | 0.0% | 0.0% |
| *Streptococcus pneumoniae* | 2 | 0.0% | 0.0% | 100.0% | 50.0% | 0.0% |  | 50.0% | 50.0% | 50.0% | 0.0% | 50.0% | 50.0% | 0.0% |
| *Streptococcus salivarius* | 2 | 50.0% | 50.0% | 0.0% | 100.0% | 0.0% |  | 0.0% | 100.0% | 0.0% | 0.0% | 50.0% | 50.0% | 0.0% |
| *Streptococcus mitis* | 1 | 0.0% | 0.0% | 100.0% | 100.0% | 0.0% |  | 0.0% | 0.0% | 0.0% | 100.0% | 0.0% | 0.0% | 100.0% |
| *Streptococcus oralis* | 1 | 0.0% | 100.0% | 0.0% | 100.0% | 0.0% |  | 0.0% | 0.0% | 0.0% | 100.0% | 0.0% | 0.0% | 100.0% |
| *Streptococcus anginosus* | 2 | 50.0% | 50.0% | 0.0% | 100.0% | 0.0% |  | 0.0% | 50.0% | 0.0% | 50.0% | 50.0% | 50.0% | 0.0% |
| *streptococcus pyogenes* | 2 | 100.0% | 0.0% | 0.0% | 100.0% | 0.0% |  | 0.0% | 100.0% | 0.0% | 0.0% | 100.0% | 0.0% | 0.0% |
| *streptococcus agalactiae* | 2 | 100.0% | 0.0% | 0.0% | 100.0% | 0.0% |  | 0.0% | 100.0% | 0.0% | 0.0% | 100.0% | 0.0% | 0.0% |
| **Total Gram-positive** | **24** | **66.7%** | **20.8%** | **12.5%** | **91.6%** | **4.2%** |  | **4.2%** | **79.2%** | **8.3%** | **12.5%** | **75.0%** | **16.7%** | **8.3%** |
| *Candida* |  |  |  |  |  |  |  |  |  |  |  |  |  |  |
| *Candida albicans* | 2 | 100.0% | 0.0% | 0.0% | NFP | NFP |  | NFP | 50.0% | 0.0% | 50.0% | NFP | NFP | NFP |
| *Candida glabrata* | 2 | 100.0% | 0.0% | 0.0% | NFP | NFP |  | NFP | 100.0% | 0.0% | 0.0% | NFP | NFP | NFP |
| *Candida tropicalis* | 2 | 100.0% | 0.0% | 0.0% | NFP | NFP |  | NFP | 100.0% | 100.0% | 0.0% | NFP | NFP | NFP |
| *Candida parapsilosis* | 2 | 100.0% | 0.0% | 0.0% | NFP | NFP |  | NFP | 100.0% | 100.0% | 0.0% | NFP | NFP | NFP |
| *Issatchenkiaorientalis* | 2 | 100.0% | 0.0% | 0.0% | NFP | NFP |  | NFP | 50.0% | 0.0% | 50.0% | NFP | NFP | NFP |
| **Total *Candida*** | **10** | **100.0%** | **0.0%** | **0.0%** | **NFP** | **NFP** |  | **NFP** | **80.0%** | **0.0%** | **20.0%** | **NFP** | **NFP** | **NFP** |
| Anaerobes |  |  |  |  |  |  |  |  |  |  |  |  |  |  |
| *Bacteroidesfragilis* | 2 | NFP | NFP | NFP | 100.0% | 0.0% |  | 0.0% | NFP | NFP | NFP | 100.0% | 0.0% | 0.0% |
| **Total Anaerobes** | **2** | **NFP** | **NFP** | **NFP** | **100.0%** | **0.0%** |  | **0.0%** | **NFP** | **NFP** | **NFP** | **100.0%** | **0.0%** | **0.0%** |
| **Overall** | **54** | **82.7%** | **9.6%** | **7.7%** | **95.0%** | **2.5%** |  | **2.5%** | **84.6%** | **5.8%** | **9.6%** | **85.0%** | **10.0%** | **5.0%** |

NFP: Not Flagged Positive

**Supplementary Table 3. Comparison of direct IH MALDI-TOF MS identification from BD BACTEC Plus Aerobic/F Culture Vials, BD BACTEC Plus Lytic/10 Anaerobic/F Culture Vials and BD BACTEC Myco/F Lytic Culture Vials.**

| Organisms | BD BACTEC^TM^ Plus Aerobic/F Culture Vials | | | BD BACTEC^TM^ Plus Lytic/10 Anaerobic/F Culture Vials | | | BD BACTEC^TM^Myco/F Lytic Culture Vials | | |
| --- | --- | --- | --- | --- | --- | --- | --- | --- | --- |
|  | Isolates identified by MALDI-TOF at the species level | Isolates identified by MALDI-TOF at the genus level | Isolates mis/not identified by MALDI-TOF | Isolates identified by MALDI-TOF at the species level | Isolates identified by MALDI-TOF at the genus level | Isolates mis/not identified by MALDI-TOF | Isolates identified by MALDI-TOF at the species level | Isolates identified by MALDI-TOF at the genus level | Isolates mis/not identified by MALDI-TOF |
| *Staphylococcus* | 96.7% (29/30) | 3.3% (1/30) | 0% (0/30) | 100% (14/14) | 0% (0/14) | 0% (0/14) | 50.0% (1/2) | 50.0% (1/2) | 0% (0/2) |
| *Streptococcus* | 71.4% (10/14) | 0% (0/14) | 28.6% (4/14) | 64.3% (9/14) | 0% (0/14) | 35.7% (5/14) | 100% (1/1) | 0% (0/1) | 0% (0/1) |
| *Entercoccus* | 100% (7/7) | 0% (0/7) | 0% (0/7) | 100% (7/7) | 0% (0/7) | 0% (0/7) | 0.0% | 0.0% | 0.0% |
| Other GP cocci | 0.0% | 0.0% | 0.0% | 100% (1/1) | 0% (0/1) | 0% (0/1) | 0.0% | 0.0% | 0.0% |
| GP Rods | 16.7 (1/6) | 50.0% (3/6) | 33.3% (2/6) | 83.3% (5/6) | 0% (0/6) | 16.7% (1/6) | 0.0% | 0.0% | 0.0% |
| **Total GP bacteria** | **82.5% (47/57)** | **7.0% (4/57)** | **10.5% (6/57)** | **85.7% (36/42)** | **0% (0/42)** | **14.3% (6/42)** | **66.7% (2/3)** | **33.3% (1/3)** | **0% (0/3)** |
| *Enterobacteriaceae* | 96.8% (60/62) | 3.2% (2/62) | 0% (0/62) | 100% (66/67) | 0% (1/67) | 0% (0/67) | 0.0% | 0.0% | 0.0% |
| Non-fermenting bacilli | 88.9% (24/27) | 7.4% (2/27) | 3.7% (1/27) | 0.0% | 0.0% | 0.0% | 80.0% (4/5) | 0% (0/5) | 20.0% (1/5) |
| Other GN bacilli | 0% (0/4) | 0% (0/4) | 100% (4/4) | 0% (0/1) | 0% (0/1) | 100% (1/1) | 0.0% | 0.0% | 0.0% |
| **Total GN bacteria** | **90.3% (84/93)** | **4.3% (2/93)** | **5.4% (5/93)** | **97.0% (66/68)** | **0% (1/68)** | **0% (1/68)** | **80.0% (4/5)** | **0% (0/5)** | **20.0% (1/5)** |
| **Anaerobes** | **0.0%** | **0.0%** | **0.0%** | **80.0% (4/5)** | **0% (0/5)** | **20.0% (1/5)** | **0.0%** | **0.0%** | **0** |
| **Yeasts** | **88.9% (8/9)** | **11.1% (1/9)** | **0% (0/9)** | **0.0%** | **0.0%** | **0.0%** | **100% (2/2)** | **0% (0/2)** | **0% (0/2)** |
| **Overall** | **87.4% (139/159)** | **5.7% (9/159)** | **6.9% (11/159)** | **92.1% (106/115)** | **0.9% (1/115)** | **7.0% (8/115)** | **80.0% (8/10)** | **10.0% (1/10)** | **10.0% (1/10)** |
